# Thyroid hormones and minerals in immunocorrection of disorders in autoimmune thyroid diseases

**DOI:** 10.3389/fendo.2023.1225494

**Published:** 2023-08-30

**Authors:** Viktor Kravchenko, Tamara Zakharchenko

**Affiliations:** Epidemiology of Endocrine Diseases, Vasily Pavlovich Komisarenko Institute of Endocrinology and Metabolism, Kyiv, Ukraine

**Keywords:** autoimmune thyroid diseases, thyroid hormones, iodine, selenium, zinc, iron, magnesium, immunomodulating effect

## Abstract

Thyroid hormones and essential elements iodine (I), selenium (Se), iron (Fe), copper (Cu), zinc (Zn), calcium (Ca), magnesium (Mg), etc. play an important role in the work of many organs and systems of the body, including the immune system and the thyroid gland, and a violation of their supply can be the cause of pathological changes in them. In pathology, the interaction between thyroid hormones (TG), minerals and the immune system is disturbed. The review of the literature examines the immunomodulatory role of TG, minerals, their properties, and their participation in the pathogenesis of autoimmune thyroid diseases (AITD). The study of the relationship between the excess or deficiency of minerals and AITD is described. The basis of the development of AITD - Hashimoto’s thyroiditis (HT), Graves’ disease (GD), Graves’ ophthalmopathy (GO) is the loss of immune tolerance to thyroid antigens - thyroid peroxidase (TPO), thyroglobulin (Tg) and thyroid-stimulating hormone receptor (TSH-R). Immune-mediated mechanisms - production of autoantibodies to thyroid antigens and lymphocytic thyroid infiltration - are involved in the pathogenesis of AITD. Insufficiency of regulatory T cells (Treg) and regulatory B cells (Breg), imbalance between Th17-lymphocytes and Treg-lymphocytes, abnormal production of pro-inflammatory cytokines has a significant influence on the progression of AITD. With AITD, the balance between oxidants and antioxidants is disturbed and oxidative stress (OS) occurs. The lack of modern effective pharmacological therapy of AITD prompted us to consider the mechanisms of influence, possibilities of immunocorrection of pathogenetic factors using TG, micro/macronutrients. In order to develop a more effective treatment strategy, as well as approaches to prevention, a critical analysis of the ways of immunotherapeutic use of dietary supplements of I, Se, Zn, Mg and other minerals in AITD was carried out.

## Introduction

Autoimmune thyroid diseases (AITD), namely autoimmune thyroiditis (AIT)/Hashimoto’s thyroiditis (HT), Graves’ disease (GD), Graves’ ophthalmopathy (GO), are complex, polygenic lesions with both similarities and differences that largely programmed by genetic factors (1, 2). The implementation of these factors depends on various environmental influences, such as stress, smoking, bacterial and viral infections, chemical pollutants, as well as dietary iodine (1, 3). AITD are one of the most common pathologies in the structure of endocrine diseases and are the most frequent cause of hypothyroidism and thyrotoxicosis. AIT begins asymptomatically, is combined with damage to thyrocytes, and the synthesis of autoantibodies is the final stage of the immune response to autoantigens TPO (thyroid peroxidase) and Tg (thyroglobulin). The prevalence of TPOAb (11.3%) and TgAb (2.0%) in euthyroid subjects was observed in 15.3% of individuals (4). The odds of TPOAb is higher in females (5). Over time, latent AIT turns into subclinical and overt thyroiditis with hypothyroidism. The incidence of manifest AIT in different countries was diagnosed from 27 to 273 cases per 100,000/year of the population (6). Among children and adolescents with HT, euthyroidism was detected in 52.1% of patients, overt or subclinical hypothyroidism in 41.4%, overt or subclinical hyperthyroidism in 6.5% (7). The prevalence of thyroid dysfunction in pregnant women is high (11%) and is 5.6% with subclinical hypothyroidism, 3.5% with overt hypothyroidism, and 1.5% with subclinical hyperthyroidism in patients observed in the 3rd trimester of pregnancy (8). The postpartum period is characterized by an exacerbation of the disease (9). AITD can occur both in isolation and in combination with other autoimmune disorders, such as type 1 diabetes, celiac disease, vitiligo, etc. (10, 11).

The prevalence of hyperthyroidism is 1.2-1.6%, of which 0.5-0.6 are obvious and 0.7-1% are subclinical (12). The most common cause of this disease is GD. The age-adjusted incidence of GD in adults in Sheffield, UK is 24.8 per 100,000/year (13). In patients with GD binding of antibodies to the thyroid-stimulating hormone receptor (TSH-R) increases intracellular cAMP production, resulting in thyroid hormone (TG) release, thyrotoxicosis, and thyrocyte hyperplasia. Graves’ Ophthalmopathy associated with GD (GO) occurs in 25-30% of cases and is its extrathyroidal manifestation (14, 15). The frequency of GO in women is higher (2.67–3.3 cases/100,000/year) than in men (0.54–0.9 cases/100,000/year) (16).

Treatment of TG of patients with HT is not effective enough. Patients with HT who received replacement with levothyroxine (L-T4) did not always achieve clinically significant results, which requires additional therapeutic agents (17). After discontinuation of antithyroid drugs, 73.6% of GD patients went into remission, but 13.3% had treatment failure and 36.7% relapsed (13). The benefit of intravenous glucocorticoids in mild GO is limited and does not justify the possible side effects. However, treatment of mild GO is necessary to prevent progression to more severe forms (16).

The significant prevalence of AITD, a long latent period and the involvement of autoimmune processes in their occurrence, development of concomitant diseases, insufficient effectiveness of the main pharmacotherapy, and a decrease in the quality of life of patients require the development of adequate approaches for the correction of immune processes in order to prevent and treat diseases. Until recently, there are no clear established effective recommendations for the treatment of AITD, and the separation of their certain manifestations from an obvious disease. The problem of treatment and prevention of AITD is of particular social importance in connection with environmental contamination, unbalanced iodine prophylaxis, the use of some drugs that act as factors of damage to the immune system, and the realization of the effect at the level of target organs, in particular the thyroid. The need for diagnosis, treatment and prevention of AITD is also due to the need to prevent concomitant diseases, including disorders of the cardiovascular system, hypercholesterolemia, atrial fibrillation, encephalopathy, others autoimmune diseases (1–3, 10, 11, 18).

## Cellular and humoral immunity in the pathogenesis of AITD

In addition to genetic, cellular and humoral immune mechanisms, loss of immune tolerance to thyroid autoantigens is the basis for the development of AITD. In patients with AITD, autoreactive lymphocytes infiltrate the thyroid parenchyma, where they recognize thyroid antigens: Tg, TPO, TSH-R, and the sodium iodide symporter (19, 20). Thyroid antigens are presented by dendritic cells (DCs), macrophages (MF) and B-lymphocytes to both maturing CD4^+^ lymphocytes and cytotoxic CD8^+^ lymphocytes (21).

In the pathogenesis of AITD, cells of both adaptive (specific) immunity - CD4^+^-, CD8^+^-lymphocytes, peripheral regulatory T-cells (pTreg), etc. and innate immunity - natural killer cells (NK-cells), neutrophils, natural Treg (nTreg), which interact and are mutually regulated, play a significant role (22–25).

The clinical manifestation of the autoimmune phenotype towards HT or GD largely depends on the balance of the immune response induced by T-helper cells (Th1 or Th2), antigen-presenting cells (APCs) and the cytokine profile that dominates at that moment in the thyroid parenchyma (26). In the pathogenesis of AITD, considerable attention is focused on the role of subpopulations of T cells - Treg and Th17 lymphocytes, as well as B lymphocytes and APCs, especially DCs (27). Cytokines that produce Th2 cause excessive stimulation of B lymphocytes and plasma cells, as a result of which the production of thyroid autoantibodies begins (22, 26).

**Figure 1:**
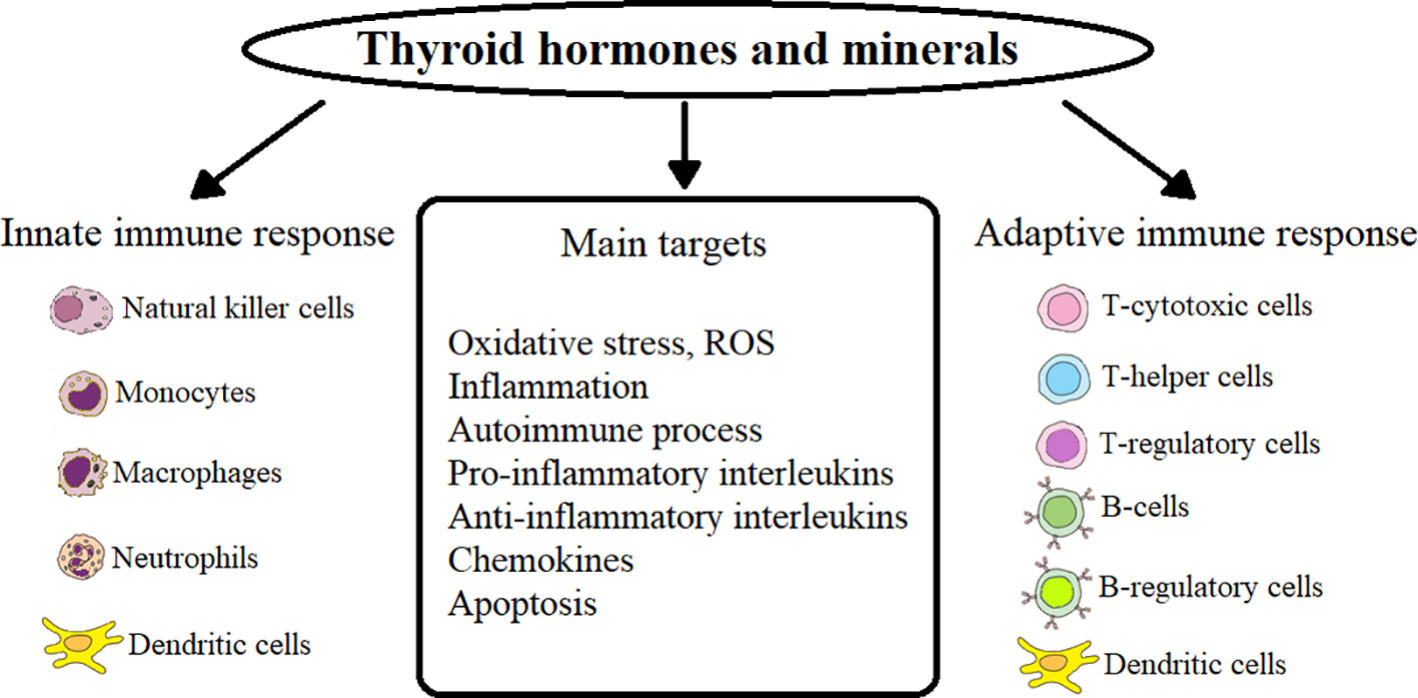
Influence of thyroid hormones and minerals on innate and adaptive immunity in AITD.

In HT patients, primary hypothyroidism occurs after the destruction of CD8^+^ and MF directly or with the help of released cytokines by NK-cells in ADCC (antibody-dependent cell-mediated cytotoxicity) of a sufficient number of follicular cells that produce TG (19, 20, 28). Accumulation of reactive oxygen species (ROS) in thyroid tissues leads to apoptosis of thyroid cells during HT (29). In HT patients, the induction of neutrophil extracellular traps (NET) increases, which depends on the accumulation of ROS through the activation of nicotinamide adenine dinucleotide phosphate (NADPH) oxidase and the production of interleukin (IL) IL-6 (30). Oxidative stress (OS) is closely related to the autoimmune process in thyroid disease (GD and GO, as well as HT) - a process that damages cellular structures, including lipids and membranes, proteins and DNA (31–33). The main points of action of TG, minerals on innate and acquired immunity and their targets are presented in **Figure 1**.

In the following text, we considered these questions in more detail.

## Thyroid status is one of the components of immunomodulating effect in AITD

In physiological and pathological conditions, the interaction between TG - 3,3’,5,5’-tetraiodo-L-thyroxine (T4) and 3,3’,5-triiodo-L-thyronine (T3) and the immune system has been established. Immunocytes can be considered as important target cells of TG, which, if necessary, can produce and deposit TSH and T3, have receptors for these hormones and related genes, which makes them capable of forming new endocrine glands (34). TG metabolism and thyroid status are related to various aspects of the immune response. TG play an important role in both innate immunity and the adaptive immune response. For the most part, a hyperthyroid state activates the immune system, while hypothyroidism leads to its suppression. The effect of TG is mediated by various factors, including nuclear factor-kappa B (NF-κB), protein kinase C and β-adrenergic receptor signaling pathways (35).

Mechanisms underlying the action of TG include both genomic (via nuclear receptors) and non-genomic (membrane) effects. Mammalian target of rapamycin (mTOR) can be an important target of the non-genomic TG pathway. With the help of the membrane receptor for TG integrin αvβ3 on neutrophils, TG induces ROS production (36). An increase in the level of thyronine leads to an increase in the pro-inflammatory reaction of neutrophils, MF and DCs (37).

Thyroid hormones play an important role both in innate immunity (neutrophils, MF, NK-cells and DCs) and in the adaptive immune response (MF, DCs, CD4^+^, CD8^+^). Triiodothyronine promotes the phenotypic maturation of DCs by regulating main histocompatibility complex-II (MHC-II) and costimulatory molecules. Functional activation of DCs stimulates NK-cells, promotes an increase in proinflammatory cytokines (IL-12, IL-6, IL-23, IL-1β) and a decrease in antiinflammatory transforming growth factor-β (TGF-β1), which control adaptive reactions, which contributes development of Th1 and Th17, IL-17-producing γδT cells and cytotoxic T cells. At the same time, the function of the Treg subpopulation decreases. Activated T3 DCs also increase the expression of the chemokine receptor CCR7, which promotes their migration to the lymph nodes, where they present absorbed and processed antigens in a complex with MHC-II to specific T cell receptors (TCR) of naïve T cells (38). A high level of serum T3 causes inhibition of Treg function, reducing the expression of suppressive membrane proteins programmed cell death protein-1 (PD-1) in patients with GD (39).

## The role of minerals in the pathogenesis of AITD. Prerequisites for their immunotherapeutic use

### Iodine

Iodine deficiency is a global problem that affects all strata of the population and exists in many countries of the world. More than 2 billion of the world’s population are at risk of insufficient iodine supply (40). The consequences of I deficiency are endemic goiter, irreversible brain damage, cretinism, hypothyroidism, and cancer of the thyroid, breast, and stomach. The main biological role of I is in the biosynthesis of TG, since I is a necessary structural component of TG (41).

In the body, I exhibits immunomodulating effect. Oxidation of I to hypoiodite (IO^-^) has significant bactericidal, antiviral, antifungal and anticancer activity. A significantly high content of I is found in leukocytes that participate in immunity and produce iodine-containing radicals. In addition, in the production of ROS by leukocytes, as in thyrocytes, the key enzyme is peroxidase. Iodine stimulates the myeloperoxidase activity of neutrophils, MF, increasing their bactericidal potential and inflammation, reduces the autophagy process. On the other hand, I also performs an antioxidant function in the body, as it is an absorber of ROS (42).

Chronic high intake of I is associated with an increased incidence of AIT. An excess of I can increase the antigenicity of Tg, stimulate the production of cytokines and chemokines, which can increase the accumulation of immunocompetent cells in the thyroid gland, increase the level of OS in thyrocytes, stimulate lipid peroxidation and damage to thyroid tissue (43). In mouse, a NOD.H-2h4 strain prone to AIT and susceptible individuals, excess I increases the number of intrathyroidal infiltrating Th17 and inhibits the development of Treg, causes abnormal expression of tumor necrosis factor-related apoptosis-inducing ligand (TRAIL) in thyrocytes, leading to their apoptosis (44, 45). The effect of excess I on thyroid follicular cells (TFCs) obtained from HT patients resulted in inhibition of autophagy, autophagy-associated protein LC3B-II (lain chain 3B-II), TGF-β1, and increased ROS production, caspase-3 activation and apoptosis of TFC, the Akt/mTOR signaling pathway (46).

In regions with excessive consumption of I, there is an increased frequency of AIT characterized by high titers of TPOAb and TgAb (47). Deficiency or excess consumption of I may serve as a risk factor for thyroid diseases. In iodine-deficient countries, iodine-deficiency diseases predominate in the structure of thyroid disease, and in regions with normal or greater iodine supply, there is an increase in thyroid disease (48). More than adequate consumption of I in the population due to a poorly controlled iodine prophylaxis program can cause euthyroid or subclinical hypothyroid AIT (49). The initiation of a 20-year (1997-2016) salt iodization program in Denmark resulted in a steady increase in the incidence of hypothyroidism among individuals living in regions of moderate and mild iodine deficiency, but only among young and middle-aged participants (50). After universal salt iodization (USI) over 16 years in 15,008 adults from 10 cities in eastern and central China, the prevalence of clinical hypothyroidism, subclinical hypothyroidism, clinical hyperthyroidism, GD, and the presence of thyroid antibodies were found to be significantly higher in cities with more than adequate consumption of I. Compared with a five-year prospective study conducted in 1999, the prevalence of goiter decreased significantly, but there was a significant increase in thyroid nodules. The prevalence of subclinical hypothyroidism, the level of TPOAb and TgAb increased significantly (51). After two decades of USI in 78,470 individuals from 31 provinces in Mainland China, the prevalence of clinical hyperthyroidism and GD was higher in women than in men, and significantly higher in those aged 30-39 compared to those aged 60 and over, and stabilized in older individuals (48).

However, in HT patients from an iodine-deficient region of Turkey, no association was found between HT and urinary I levels, which were not elevated compared to the general population. Also, no significant difference was found between the levels of fT3, fT4, ТPОАb, TgАb and thyroid gland volume in individuals with urinary I concentration above and below 100 μg/L. The difference reached a probable level only when comparing indicators with TSH (52). A national cross-sectional survey in mainland China found that after two decades of USI, the prevalence of TPOAb remained low. More than sufficient intake of I had an inverse relationship with TPOAb, and suggests that UIC from 100 to 299 μg/L is optimal and safe for the occurrence of AITD (53). Moreover, a multivariate regression analysis of a case-control study by Attard CC. et al, 2022 (5), showed that a higher intake of iodine-rich foods reduced the odds of TPOAb production (OR 0.864, 95% CI 0.761-0.981; p=0.024).

Growth in the prevalence of AITD should not limit the introduction of I supplements in the general population. Consumption of I in a concentration not exceeding 300 μg/L is safe with regard to AITD and does not increase the risk of autoimmune disorders for the population. Studies of the long-term consequences of the use of I on the occurrence and development of AITD clearly demonstrated that the early increase in thyroid antibodies is mostly transient, differs between populations under the influence of genetic and environmental factors, and does not always coincide with the presence of AITD or its further development (54). Excess I, which can disrupt thyroid function, in part, through OS, is much less harmful because it affects only a small percentage of individuals susceptible to AITD. While iodine deficiency affects the population and causes endemic consequences (55).

Today, new strategies for the diagnosis and treatment of iodine deficiency are attracting attention, focusing on the potential role of myo-inositol in combination with I (56). Myoinositol plays a crucial role in the functioning of the thyroid gland and AITD, as it regulates the organification of I and the biosynthesis of TG through the formation of hydrogen peroxide (H_2_O_2_) in thyrocytes (57). Combined supplementation of I and myoinositol may contribute to increased availability of I in thyrocytes, improving thyroid function (58).

### Selenium

The normal function of the thyroid gland depends on a variety of trace elements for the synthesis and metabolism of TG, primarily I and Se. Selenium is a cofactor for enzymes that are present in thyroid cells, such as iodothyronine deiodinase (DIO), which catalyzes the conversion of T4 to T3, thioredoxin reductase (TR), which is involved in the regulation of the redox state, and glutathione peroxidase (GPx), which catalyzes the reduction of H_2_O_2_ to H_2_O, using reduced glutathione (GSH) as a cosubstrate. In physiological conditions, selenoproteins are an effective defense system against a large amount of ROS, which thyrocytes constantly generate on their surface in order to accept electrons from oxidative reactions (59). In the process of iodination of tyrosyl residues and TG synthesis, H_2_O_2_ is a necessary substrate for TPO. Thyroid H_2_O_2_ production is stimulated by myoinositol, which indirectly controls the inositol-dependent signaling pathway of TSH (56, 57).

Selenoproteins are important for immunomodulating effect. In mice, a high-Se diet increased TCR signaling in CD4^+^ T cells, increased IL-2 expression, shifted the Th1/Th2 balance toward a Th1 phenotype, higher levels of interferon-γ (IFN-γ) and CD40 ligand (60). In physiological conditions, selenoproteins participate in the activation of proliferation, differentiation and redox metabolism of T cells. At the same time, Se is involved in reducing excessive immune reactions and chronic inflammation (61). Through molecular mechanisms, selenoproteins regulate T-cell effector function related to redox signaling, calcium (Ca^2+^) flux, and OS (62).

Selenoproteins prevent the excessive formation of ROS, which can lead to autoimmune diseases or chronic inflammation. Decreased serum Se concentration may lead to chronic OS through neutrophil ROS production (63). Selenium deficiency reduces the expression and activity of these antioxidant enzymes (64).

However, excessive production of ROS is involved in various stages of the pathogenesis of thyroid diseases and activates possible common triggers of GD and HT, differentiated thyroid cancer and even endemic goiter (31, 64). Deficiency of Se or its excess with dysfunction of mitochondria (organelles that contain selenoproteins) leads to thyroid disorders (65). Thyrocytes and thyroid fibroblasts pre-incubated with selenocompounds (selenomethionine and selenite) showed a dose-dependent increase in viability and a decrease in apoptosis indicators (caspase-3 activity, BAX mRNA levels) and an increase in the level of the inhibitor of apoptosis (BCL-2 mRNA) after incubation with H_2_O_2_, and also caused an increase in GPx activity (66).

Selenium together with I play a key role in the development of AITD (67). There is a relationship between insufficient intake of Se and AITD. Epidemiological data indicate the prevalence of AITD with Se deficiency (68). Although some authors did not note the relationship between the prevalence of HT and Se content (69).

In patients with GD, an inverse relationship of serum Se levels with T4 and TSH hormones and an inverse relationship with T3, T4, and TSH hormones were found in patients with GO, although there was no significant difference in Se levels in patients compared to healthy individuals (70). However, the results of other studies recorded a significant decrease in the level of Se in the serum of patients with newly diagnosed GD and autoimmune overt hypothyroidism (71). Deficiency of Se in the serum of patients with GD increased with age and was correlated with an increased titer of antibodies to the TSH receptor (TRAb). In addition, a relationship between the level of Se and ioduria was registered in patients with GD (67). A decrease in serum Se levels in HT and especially in GD and GO is associated with the pathogenesis of the disease (72).

Treatment of HT remains symptomatic and is based on taking, as needed, synthetic TG to correct hypothyroidism. Surgery is performed when the goiter is large enough. The value of Se and the presence of its deficiency in AITD argue for the need to replenish it in the body.

The formation of TPOAb and TgAb is most often associated with HT, while the activation of TRAb formation is most often associated with GD, although their overlap has been recorded. There is conflicting information regarding the effect of Se supplementation on TPOAb and TgAb levels in HT patients. Most authors reported a decrease in TPOAb and TgAb levels after using different doses of Se (selenomethionine) supplements with or without L-T4 (73, 74). TPOAb titers decreased to varying degrees in patients with different genotypes of the single nucleotide polymorphism r25191G/A, which indicates interindividual differences (75). The results of twenty-three studies on the efficacy of Se with or without L-T4 in adults with HT compared with placebo and/or L-T4 showed that populations that did not receive L-T4 but received Se had lower levels of TPOAb, TgAb for a short time. At the same time, significant changes in the TSH level were not observed (76).

Current data do not always justify the use of Se in the treatment of HT, despite the fact that it leads to a decrease in the level of TPOAb and TgAb. Perhaps this is due to the fact that autoantibodies are not a key link in the pathogenesis of HT, they begin to be produced already in response to damage to thyrocytes and are the result of an autoimmune response. There is an assumption about the indirect participation of modified autoantibodies (desialylated IgG N-glycans) in ADCC of NK cells and mononuclear lymphocytes involved in the destruction of thyrocytes (28). Despite the fact that HT is a chronic autoimmune process, and the production and circulation of autoantibodies in the peripheral blood is long-term, the therapeutic reduction of TPOAb and TgAb levels in HT is not conclusive evidence for the use of Se drugs.

The results of research on the use of selenium are conflicting. Se supplementation is necessary in Se-deficient individuals, but may have some health risks in Se-excessive individuals, including diabetogenic effect. Addition of Se to L-T4 therapy may be appropriate in patients with low Se intake and mild or early stage HT. Se supplementation is well tolerated but should not be universally recommended (59). Done by Winther K.H. et al, 2017 (17) systematic review of eleven publications covering nine controlled trials and a meta-analysis of case-effect models found no effect of Se supplementation on TSH and quality of life. Individuals receiving Se supplementation but not receiving L-T4 replacement had no changes in thyroid ultrasound findings, and individuals receiving L-T4 replacement occasionally showed clinically significant findings. Available research data do not support the use of routine Se supplementation in the treatment of patients with HT or GD. However, correction of moderate and severe Se deficiency may be an advantage in the prevention and treatment of these disorders.

The effect of Se on immunological indicators may depend on its dose and the stage of HT development (hyperthyroid, euthyroid, hypothyroid). Se concentration in serum decreases in inflammatory conditions and may vary depending on the severity and duration of the inflammatory process. The immunomodulating effect of Se on the humoral and cellular components of the autoimmune process in the thyroid gland is noted. In rats with experimental AIT, after the use of selenium yeast dietary supplement, in addition to a decrease in the levels of TgAb, TPOAb and a decrease in B-cell-activating factor (BAFF) in the blood serum, the expression of IL-10 in the thyroid tissue increased, the number of Breg in the spleen increased, and also decreased TSH level (77). Selenium increased the percentage and enhanced the activity of Treg, suppressed the secretion of pro-inflammatory cytokines, prevented the apoptosis of follicular cells and provided protection against thyroiditis (45, 74). Treatment of patients with HT L-T4 and capsule with Se yeast for 3 months not only reduced the level of autoantibodies, but also the level of IL-2, which indicates the suppression of the inflammatory process (73).

The main proposed therapeutic mechanisms of AITD include increasing the activity of GPx and TR enzymes in blood plasma and reducing toxic concentrations of H_2_O_2_ and lipid hydroperoxides. As part of selenoproteins, Se takes an active part in antioxidant, redox and anti-inflammatory processes (59). Treatment of HT patients with selenium yeast tablets for 6 months significantly increased the level of Se, GPx3, and selenoprotein P1 (74). In patients with primary hypothyroidism, after treatment with L-T4 and Se, the level of the OS marker - malondialdehyde (MDA) decreased (78). Se supplementation in euthyroid, subclinical, or overt hypothyroid patients not only reduced thyroid autoantibodies, lowered or maintained TSH, decreased fT4/fT3 ratio, but also decreased OS and inflammation, and improved quality of life, structure, and volume thyroid gland (79). The use of antioxidant nutrients, including Se, reduces OS and thyroid damage in patients with HT (33).

Thyroid function in patients with HT and hypothyroidism may be favorably influenced by myoinositol, which regulates the formation of H_2_O_2_ in thyrocytes. Insufficiency of myoinositol or dysfunction of the inositol-dependent signaling pathway of TSH action can cause the development of hypothyroidism (56). The combined use of selenomethionine and myoinositol in euthyroid patients with HT reduced the level of TSH, antithyroid antibodies 6 and 12 months after treatment, and the level of TSH decreased earlier than in the group treated with selenomethionine alone (80). Moreover, the immunomodulating effect of the combination of selenomethionine and myoinositol in HT was confirmed by the fact that after treatment the level of the chemokine CXCL10, which is a potent chemoattractant for activated T-lymphocytes, also decreased, suggesting the possibility of reducing the risk of progression of euthyroidism to hypothyroidism (81).

Selenium supplementation can be offered to patients with GD only if Se deficiency is confirmed. Thus, in a randomized clinical trial conducted in selenium-sufficient GD patients treated with methimazole, no beneficial effect of Se on the short-term control of thyroid hyperfunction was observed (82). A randomized clinical trial by Calissendorff J. et al, 2015 (83) showed that Se has a beneficial effect in a cohort of hyperthyroid and Se-deficient patients treated with methimazole and L-T4 under a block-and-replace regimen. A meta-analysis by Zheng H. et al, 2018 (84) of ten randomized placebo-controlled trials involving 796 patients showed short-term (3 months) efficacy of Se supplementation on fT4 and fT3 levels in patients with GD. TSH levels were higher in the Se group than in the control group at 3 and 6 but not at 9 months. TRAb level decreased, thyroid function improved more often after 6, but not after 9 months.

Oxidative stress plays a significant role in the pathogenesis of GO and GD (the balance between oxidants and antioxidants is disturbed in favor of ROS), and an antioxidant approach is proposed for treatment (32, 85, 86). In a primary culture of orbital fibroblasts from GO patients and control subjects treated with H_2_O_2_ to induce OS, the antioxidants vitamin C, N-acetyl-L-cysteine, melatonin, retinol, β-carotene and vitamin E were found to reduce the manifestations OS (proliferation of fibroblasts, the level of hyaluronic acid, the level of tumor necrosis factor-α (TNF-α), IFN-γ and IL1-β, which provided the basis for the possible clinical use of these substances in patients with GO (87). In patients with newly diagnosed hyperthyroidism, mean serum total antioxidant capacity (TAC) was low and MDA was high. Hyperthyroid patients treated with a combination of carbimazole and an antioxidant had significantly lower MDA, than hyperthyroid patients treated with carbimazole alone (86).

Treatment for GO, which is an extrathyroidal manifestation of GD, includes intravenous glucocorticoids and orbital radiation therapy and is generally offered to patients with moderate to severe GO. In contrast, patients with mild GO are usually treated only with local methods (16). In patients with moderate GO, there is clear evidence of a beneficial antioxidant effect of Se, from a large randomized clinical trial conducted by the European GO Group – EUGOGO. Based on EUGOGO guidelines, patients with moderate GO may be offered a 6-month course of Se. The use of Se significantly reduces eye damage and slows the progression of the disease in patients with mild GO. Although, serum Se levels were not measured in the EUGOGO study (88). Se can cause toxicity if it is administered in very high doses. A dosage of up to 200 μg/day in Se-deficient patients, or 166 μg/day in Se-sufficient patients is considered quite safe (83).

In mild GO, Se supplementation is associated with reduced disease activity as well as improved quality of life. Further studies are needed to clearly understand the biological effects of Se supplementation, considering baseline Se levels, selenoprotein gene polymorphisms, comorbidities, and main clinical outcomes (89). Results of a meta-analysis conducted by Bednarczuk T. et al, 2020 (90), who included ten prospective randomized clinical trials, suggest that Se supplementation can enhance euthyroid recovery in hyperthyroidism and GO. However, the use of Se and the determination of its effect on the level of hormones or autoantibodies requires careful conclusions due to the lack of analysis of long-term clinical observations, such as the frequency of remission after treatment with antithyroid drugs.

### Iron and copper

Deficiency of Fe and I remains the main health care problem, affecting about 30% of the world’s population (91). Iron ions are included in large quantities in the composition of proteins - hemoglobin, myoglobin, enzymes, etc. Many enzymes of DNA replication and repair (helicases, nucleases, glycosylases, demethylases) and ribonucleotide reductase use Fe as an indispensable cofactor for functioning (92).

The bioavailability of Fe controls complex metabolic processes in immune cell homeostasis and inflammation (93). Iron in the plasma has an immunomodulating effect on innate immunity by controlling the ratio of monocytes to neutrophils and neutrophil activity. Hypoferremia alters neutrophil functions, inhibiting antibacterial mechanisms but enhancing mitochondrial ROS-dependent NETosis associated with chronic inflammation (94). Iron affects the polarization of MF (M1/M2). Iron imunomodulates the termination of the inflammatory response, as elevated Fe level increase the M2 phenotype and reduce the pro-inflammatory M1 response, reducing the IL-12/IL-10 balance. Fe loading of MF *in vitro* reduces the percentage of M1 cells expressing costimulatory molecules CD86 and MHC-II, prevents lipopolysaccharide-induced pro-inflammatory response by reducing nuclear translocation of NF-κB p65 with reduced expression of, IL-1β, IL-6, IL-12 and TNF-α (95).

In blood plasma, Cu binds to ceruloplasmin, a transport protein that is a reactive protein of the acute phase. Copper stimulates the function of innate and adaptive immunity and is required to perform protective functions of the immune system (96, 97). Deficiency of any of the minerals such as Se, I, Zn, Cu, Fe, etc. can temporarily reduce immune competence or even disrupt the regulation of systemic inflammation (61). With severe Cu deficiency, the number and ability of neutrophils to generate O_2_ and kill absorbed microorganisms, T-cell proliferation and IL-2 production decrease (96).

Redox-active ions of metals such as Fe^2+^ and Cu^+^ are the most important transition metals in cells in an inert form. Oxidized forms of metals (Fe^3+^, Cu^2+^) are quickly restored in the cytosolic environment, fueling the vicious circle. To bind redox metals Fe^2+^ and Cu^+^ and prevent H_2_O_2_ from giving ^•^OH in the presence of ions of these metals in the body, there are highly active enzymes of antioxidant protection - catalase and superoxide dismutase (SOD) (98). Moreover, Cu serves as an important cofactor for enzymes such as CuSOD, cytochrome C oxidase (99). In addition to Cu, SOD subunits include trace elements Zn and manganese (Mn). Cu, ZnSOD protects the vascular and immune systems from the harmful effects of ROS. In the peripheral blood, Fe ions are in a bound form with transferrin, ferritin, lactoferrin, ceruloplasmin, which are Fe^2+^ chelators and perform an antioxidant function.

In healthy individuals with adequate intake of I the concentration of Cu and Se in the blood is significantly related to TSH and fT4 (100). Deficiency or excess of Se, I, Zn, as well as Fe and Cu affects the synthesis of TG and can disrupt thyroid homeostasis (101). Copper levels were associated with increased fT4 and tT4 levels in men. In women, Cu levels were associated with elevated tT3 and tT4 levels. The level of Cu was 20% lower in men than in women (102).

The ratio of Cu and Se may directly affect thyroid function in patients with HT and overt hypothyroidism, which may be relevant for lifelong replacement therapy during L-T4 reduction (103). Observational and controlled studies have shown frequent micronutrient deficiencies in patients with HT (104). Patients with or without GD and GO who had low serum Se levels showed relatively high serum Cu levels in GD patients, while patients with GD and GO conversely had low serum Cu levels (72).

Patients with AITD often suffer from Fe deficiency, as autoimmune gastritis, which reduces Fe absorption, and celiac disease, which causes Fe loss, are frequent comorbidities (11). In patients with HT in the stage of subclinical hypothyroidism, living in a densely populated area in the I region of Ankara, Turkey, the basal levels of Se and Fe were significantly lower than in the control group (105). Both hypothyroid and hyperthyroid patients had higher odds of Fe deficiency anemia (106).

Іron deficiency impairs thyroid metabolism. TPO is a glycosylated hemoenzyme that becomes active only after binding heme, a non-protein prosthetic group containing an Fe^2+^ ion. Іron deficiency, which corresponds to a serum ferritin level <15 μg/L, is associated with a higher prevalence of TPOAb but not TgAb in pregnant women during the first trimester and in nonpregnant women of childbearing age with subclinical hypothyroidism (107). TPOAb and TgAb positive patients with HT had significantly lower serum Fe and Mg concentrations (108).

Two-thirds of women with persistent symptoms of hypothyroidism, despite appropriate L-T4 therapy, show improvement in symptoms after recovery of ferritin (the main intracellular Fe^3+^ depot) in serum above 100μg/L (109). Double enrichment of salt I and microencapsulated Fe is suggested for effective prevention of Fe deficiency and thyroid dysfunction (110).

### Zinc

Zinc homeostasis is tightly controlled by the coordinated activities of Zn transporters and metallothioins that regulate Zn transport, distribution, and binding. Zinc acts as a signaling molecule, facilitating the transduction of various signaling cascades in response to extracellular stimuli (111).

Zinc is necessary as a catalytic, structural and regulatory ion. Immunomodulating effect of Zn is involved in immune responses, OS, apoptosis and aging. Metallothioins are protective against OS, exposure to toxic metals, infections and insufficient Zn content in food (112). Zinc is a key component of the antioxidant defense network that protects membranes from Fe^2+^-induced lipid oxidation due to Zn’s ability to prevent Fe^2+^ from binding to the membrane (113). Experimental studies *in vitro* and *in vivo* have provided evidence that Zn acts as an antioxidant by inhibiting the oxidation of macromolecules such as DNA/RNA and proteins, as well as the inflammatory response, which leads to a decrease in ROS production (114).

Zinc is associated with thymus function, which provides a unique microenvironment for the proliferation, differentiation, maturation and release of naïve T cells. Zinc is necessary for inducing the biological activity of the thymus hormone thymulin (Zn-FTS), a peptide synthesized by thymus epithelial cells (TECs). Zinc with the help of structural “zinc finger motifs” plays a key role in cell proliferation, apoptosis and reactivation of thymulin. Through the NO pathway, Zn is necessary for the biological action of arginine (115). TECs activated by thymulin perform a positive selection of immunocompetent T cells capable of recognizing foreign antigens in the context of their own MHC molecules. Deficiency of Zn^2+^ ions can reduce peripheral biological activation of thymulin.

Zinc participates in the process of physiological (age-related) involution of the thymus, which plays an important role in the immunosenescence of the body and the emergence of age-related diseases - susceptibility to infections, cancer, and autoimmune diseases. With age, the level of Zn in serum or blood plasma decreases. Low bioavailability of Zn ions due to an increase in metallothiones, IL-6 can provoke complete involution of the thymus during aging and the possible appearance of autoimmune diseases (115). Immunosenescence is perceived as a slow autoimmune process controlling the thymus. Predominance of Zn deficiency in the elderly can lead to impaired function of cells of the innate immune system and contribute to immunosenescence (116).

Due to the impaired of intrathymic expression of autoantigens (TSH-R, Tg, TPO), a loss of tolerance to these thyroid peptides is possible in genetically determined individuals with AITD under the influence of environmental trigger factors. Tolerance to self-antigens in the thymus depends on the expression of promiscuous gene expression (pGE) tissue-restricted antigens (TRA) TECs genes, which is controlled by autoimmune regulator (Aire) and forebrain embryonic zinc fingerlike protein 2 (Fezf2) regulators, which contains Zn (117).

Autoimmune diseases are associated with pathologically altered Zn levels, which provoke a change in signal transduction, which leads to impaired immune response, cell differentiation and function (118). Zn deficiency suppresses both innate and adaptive immune responses. Zn homeostasis is significantly influenced by the activation of T cells, as well as the polarization of Th cells into different subpopulations - Th1, Th2, Th17, Treg (116). The level of intracellular free Zn is important for the maintenance of Treg phenotype and function, as Zn deficiency promotes a pro-inflammatory immune response. The study of Th cells and Treg in an allogeneic model of the disease “graft against the host” demonstrated that Treg, unlike Th, had a significantly increased level of intracellular free Zn (118).

Zinc plays a significant role in the expression of melatonin receptor genes on the cell membrane of thymocytes (115). Biosynthesis and secretion of melatonin, which is synthesized mainly in the pineal gland, reciprocally affects thyroid function. Melatonin suppresses thyroid function depending on the change in the light regime (119). Nutraceuticals Se, 1-carnitine, myoinositol, resveratrol and melatonin may be effective preventive and therapeutic means AITD (120). Melatonin may play an important role in reducing ROS-mediated diseases, since one of the main functions of melatonin is to scavenge ROS (121). In patients with GO, it is possible to use antioxidant substances, including melatonin, to reduce the manifestations of OS, as evidenced by experimental studies of orbital fibroblast culture from patients with GO treated with H_2_O_2_ (87). However, there are no controlled studies that would substantiate the use of melatonin to improve thyroid function (120). Data discrepancy may be associated with melatonin receptor gene polymorphism in GD (122).

Zinc plays a key role in TG metabolism, in particular, by regulating the activity of deiodinases, thyrotropin releasing hormone (TRH) and TSH enzymes, as well as modulating the structures of the main transcription factors involved in TG synthesis (123). Cross-sectional and controlled studies showed a relationship between Zn deficiency and TG levels (124). A study by Mahmoodianfard S. et al, 2015 (125) provided some evidence for the effect of Zn alone or in combination with Se on thyroid function in hypothyroid and overweight or obese patients. Thus, mean fT3 and fT4 were significantly increased and mean serum TSH significantly decreased in the Zn+Se group (30 mg Zn as Zn gluconate and 200 μg Se as high-Se yeast) over 12 weeks, although there was no found significant changes for tT3, fT4, fT3 or TSH between groups.

In chronic diseases, including autoimmune diseases, aging, Zn deficiency can negatively affect immunological status, increase OS, and lead to the generation of pro-inflammatory cytokines (126). Zinc and vitamin D are the most promising candidates for therapeutic intervention aimed at balancing rather than suppressing the immune system (127). In HT, the use of trace elements Zn and Se is proposed to reduce oxidative damage to the thyroid gland (33).

### Calcium and magnesium

In addition to microelements I, Se, Fe, Cu, Mn, Zn, macroelements such as Ca and Mg are related to thyroid function. The interaction of Ca and Mg is important in the human body. The optimal ratio of Ca to Mg is approximately 2.0, and ratios <1.7 and >2.8 may be harmful (128).

An increase in the flow of Ca^2+^ is necessary for signal transduction, the formation of immune synapses, and the implementation of specific cytotoxicity of CD8^+^ T cells, which involves the costimulatory molecule of the cell surface LFA-1 and Mg^2+^ (129). A change in the regulation of Ca^2+^ in lymphocytes disrupts the control of metabolism, proliferation, differentiation, secretion of antibodies, cytokines and cytotoxicity, which leads to autoimmune and inflammation diseases (130). Mitochondrial intracellular Ca^2+^ is a key regulator of cell apoptosis. It has been experimentally shown that Ca uptake and mitochondrial metabolism underlie Treg survival in OS conditions (131). With the help of Ca^2+^ signaling, BAFF activates the mTOR pathway and the proliferation and survival of B-lymphocytes as producers of autoantibodies (132). The main mechanism of increasing intracellular Ca^2+^ is Mg^2+^ deficiency (133).

Magnesium exhibits immunomodulating effect. Experimental studies have shown that Mg^2+^ is a cofactor for the formation of C3 convertase, which is involved in the proteolytic activity of complement components, as well as in the binding of antigen to MF and the response to cytokines, the adhesion of Th and B cells, the synthesis of immunoglobulins, the binding of IgM with lymphocytes, ADCC (134). As a secondary signaling messenger, Mg^2+^ is involved in the activation of T cells (129, 133). Magnesium regulates the active site of IL-2-induced T cell kinase (ITK) during the CD8^+^ T cell response to viral infection in mice (135). At the same time, Mg^2+^ regulates anti-inflammatory and antioxidant reactions. Cultivation of lipopolysaccharide- or TNF-α-stimulated mesenchymal stem cells of C3H/10T1/2 mice with 5 mM Mg^2+^ increased the proliferation rate, decreased the levels of pro-inflammatory IL-1β and IL-6, and increased the levels of anti-inflammatory IL-10 and prostaglandin E2 (136).

Magnesium deficiency can cause immunodeficiency, increased acute inflammatory response, reduced antioxidant response and OS (133). Magnesium deficiency is associated with low-grade chronic inflammation involving the proinflammatory cytokines TNF-α and IL-1, the messenger IL-6, as well as E-selectins, intracellular adhesion molecule-1 (ICAM-1), and vascular cell adhesion molecule-1 (VCAM-1), acute-phase reactants - C-reactive protein and fibrinogen (137).

Magnesium regulates the proper functioning of other nutrients, such as vitamin D, which regulates the homeostasis of Ca and phosphorus (P) (133). Calcium, diacylglycerol, cAMP and various phosphate derivatives of myoinositol (phosphatidylinositol and others) are essential secondary messengers involved in the regulation of intracellular signaling cascades, in response to the activation of the TSHR, and are necessary for the production H_2_O_2_ and TG synthesis (56).

In patients with latent HT with a high titer of TPOAb and the presence of mild iodine deficiency, the content of Ca, Mg and Zn in blood serum decreases (138). Very low serum Mg is associated with an increased risk of TgAb, HT, and hypothyroidism. The risks of hypothyroidism and subclinical hypothyroidism in the lowest Mg group (≤0.55 mmol/L) were higher than in the normal Mg group (0.851-1.15 mmol/L, p<0.01, OR=4.482-4.971) (139). Significantly lower serum concentrations of Mg and Fe were found in HT patients with TPOAb and TgAb (108).

Thyroid diseases play an important role in mineral metabolism, in particular, they affect the mineral density of bone tissue. Thyroid hormones are involved in the metabolism of Ca and P. In hypothyroid patients mean serum Ca and Mg levels were decreased and P levels were elevated. Among cases of hypothyroidism, a statistically significant negative correlation was observed between Ca, Mg, and TSH, while a positive correlation was observed between P and TSH (140). In hypothyroidism, the level of Ca is lower and the level of P is higher in serum than in subclinical hypothyroidism (141). In blood serum of patients with hypothyroidism, the level of Ca was significantly reduced and the level of P was significantly increased (142). In hyperthyroidism, on the contrary, the serum Ca content increases. The average value of the levels of both Ca and P in the blood serum of patients with hyperthyroidism is significantly (p<0.001) higher, which may increase the risk of secondary osteoporosis and bone fractures. In patients with hyperthyroidism, the prevalence of hypercalcemia was 38% (143). In hyperthyroidism can be acquired impaired mitochondrial function due to deficiency of Mg, Se and antioxidant coenzyme Q10 as signs of an inflammatory process associated with changes in the musculoskeletal system (144).

For the prevention of autoimmune diseases, the reduction of excessively induced BAFF aggressive B-lymphocytes can be used to influence the intracellular level of Ca^2+^ and mTOR (132). Magnesium supplementation is a potential way to reduce inflammation and OS. Magnesium supplements reduce the levels of NF-κB, IL-6 and TNF-α, improve mitochondrial function and increase the content of antioxidant GSH (133). It is possible to improve the function and morphology of the thyroid gland after correcting exposure to stress factors together with selenomethionine and Mg citrate supplements, which showed a direct correlation between whole blood Se and serum Mg in individuals without thyroid disease and in menopausal women, whereas it was reversed in cases of hyperthyroidism (144). Supplementation of Mg, as well as Zn and vitamin A, may have beneficial effects in patients with hypothyroidism and diseases associated with hyperthyroidism. In patients with hypothyroidism, a significant increase in serum fT4, a decrease in anthropometric indices and lower levels of serum hs-CRP were found after the intervention at 10 weeks (145).

We summarized the results of the review of existing publications on the immunomodulating effect of TG and minerals on the immune system in autoimmune thyroiditis and Grave’s disease, and have presented these data in **Table 1**, where the main effects and mechanisms have been formulated.

**Table 1:** The immunomodulating influence of thyroid hormones and minerals on immunological target cells and their function in the physiological norm and in AITD.

TG/minerals	Normal/AITD	Immunological targets and effects	Corresponding references
TG	Normal GD	> neutrophils, MF, DCs, NK-cells, CD4^+^, CD8^+^, Th1, Th17, ROS, IL-12, IL-6, IL-23, IL-1β, IL-17, CCR7; < Treg < Treg, PD-1	(36–38) (39)
I	Normal *In vitro* *In vivo*/HT	> neutrophils, MF, ROS, inflammation; < ROS > ROS, OS, apoptosis > Th17, TRAIL, ROS, OS, apoptosis; TPOAb, ТgAb; < Treg, TGF-β1 </= TPOAb	(42) (43, 46) (44, 45, 47, 51, 55, 59) (53, 5)
Se	Normal *In vitro* *In vivo* (EAT) HT, GD/GO HT HT, GD GD	> CD4^+^, Th1, IL-2, IFN-γ, GPx, TR; < ROS, OS, inflammation > GPx, < apoptosis > IL-10, Breg; < BAFF; < TPOAb, ТgAb < IL-2, TPOAb, ТgAb > Treg, GPx, TR, SePP1; < OS, CXCL10, < MDA, OS < TRAb	(60–62) (66) (77) (73–75) (79, 33, 81) (78, 86) (84, 67)
Fe	Normal *In vitro* HT	> ROS NETosis, MF < МF1, IL-12/IL-10, IL-1β, IL-6, IL-12, TNF-α; > MF2 < TPOAb, TgAb	(94) (95) (107, 108)
Cu	Normal	> neutrophils, ROS, T-cells, IL-2, inflammation > CuSOD	(96, 97) (99)
Zn	Normal *In vitro*, *In vivo* HT	> Т-cells, polarization Th - Th1, Th2, Th17, > Treg < OS, ROS < OS, hs-CRP	(116–118) (114) (33, 145)
Mg	Normal *In vitro* HT	> MF, Тh, В-cells, NK-cells, CD8^+^ Т < NF-κB, IL-6, TNF-α, OS; > GSH < TNF-α, IL-1, IL-6, E-selectins, ICAM-1, VCAM-1, hs-CRP > IL-10, prostaglandin Е2; < IL-1β, IL-6 < TPOAb, TgAb, hs-CRP	(129, 134, 135) (133) (137) (136) (108, 139, 145)
Ca	Normal	> CD8^+^ Т, apoptosis, inflammation < Treg > BAFF, В-cells	(129) (131) (132)

Normal – at the physiological norm, In vitro – cell culture, In vivo – animal models, > - increase in effect, < - decrease in effect, </= - decrease/no change

## Conclusion

There is currently no effective pharmacological therapy for AITD. Treatment of AITD remains symptomatic and is based on taking, if necessary, synthetic TG to correct hypothyroidism or thyrostatics for thyrotoxicosis. The significant prevalence of AITD and the involvement of autoimmune processes in their occurrence require the development of adequate approaches for the correction of immune processes in order to prevent and treat diseases.

Replacement use of TG in case of subclinical and obvious hypothyroidism and inhibition of their synthesis in thyrotoxicosis is appropriate for normalizing thyroid status, but is insufficient for reducing inflammation and OS, restoring the immune system, and suppressing the autoimmune process in the target organ in AITD. Deficiency or excess consumption of I may serve as a risk factor for thyroid diseases. Careful monitoring of iodine prophylaxis is recommended to avoid the consequences of both deficiency and excess I. Addition of Se preparations to L-T4 may be appropriate in patients with low Se and mild or early stage HT. In patients with moderate GO, there is clear evidence of a beneficial effect of Se from a large randomized clinical trial conducted by the European GO Group – EUGOGO.

For the treatment of GD, GO and HT, an antioxidant approach is possible with the use of bio-supplements of elements (I, Se, Zn, Fe, Cu and Mg) as an adjunct in patients with their deficiency. The possibilities of the immunotherapeutic effect of minerals, in addition to the level of thyroid autoantibodies, can be aimed at OS markers. The main therapeutic mechanisms of AITD are the reduction of OS, the inflammatory process that damages thyrocytes. The targets of immunocorrection with the participation of minerals (I, Se, Zn, Fe, Cu, and Mg) in AITD can be the normalization of the function of neutrophils as producers of excess ROS and inflammation, reduction of the secretion of pro-inflammatory cytokines Th17 and chemokines, activation of Treg and their cytokines as the main inhibitors of inflammation and autoimmune process.

Determining the immunomodulating effect of TG, minerals, and researching the possibility of using them as means of immunocorrection, taking into account the pathogenesis of AITD, can contribute to the development of a more effective treatment strategy, as well as approaches to prevention.

## Author contributions

VK and TZ conceived and designed the review. The manuscript was written by TZ, VK. All authors have read and approved the published version of the manuscript.
